# Toxoplasmosis in Zoo Animals: A Retrospective Pathology Review of 126 Cases

**DOI:** 10.3390/ani12050619

**Published:** 2022-03-01

**Authors:** Daniela Denk, Simon De Neck, Shannon Khaliq, Mark F. Stidworthy

**Affiliations:** 1 Institute of Veterinary Pathology, Centre for Clinical Veterinary Medicine, Ludwig-Maximilians-University, 80539 Munich, Germany; 2 Division of Pathology, Department of Biomolecular Health Sciences, Faculty of Veterinary Medicine, Utrecht University, Yalelaan 1, 3584 CL Utrecht, The Netherlands; s.m.deneck@uu.nl; 3 Bristol Veterinary School, Langford House, Bristol BS40 5DU, UK; sk12727.2012@my.bristol.ac.uk; 4 International Zoo Veterinary Group, Parkwood Street, Keighley BD21 4NQ, UK; m.stidworthy@izvg.co.uk

**Keywords:** *Toxoplasma gondii*, zoo animals, systemic pathology review, ring-tailed lemur, slender tailed meerkat, Pallas’ cat, squirrel monkey

## Abstract

**Simple Summary:**

*Toxoplasma gondii* is widespread amongst domestic animals and can affect humans. Whilst the disease is of economic importance and well-studied in ruminants, this is the first large-scale study evaluating toxoplasmosis in zoo animals. The aim of this study was to identify species that are particularly susceptible to disease, review clinical symptoms, organ distribution and key target tissues, highlight unusual outbreaks/presentations, assess predilections, evaluate toxoplasmosis as the cause of death and consider seasonality. A total of 31 species were represented, with ring-tailed lemurs (RTLs), meerkats, Pallas’ cats and squirrel monkeys most affected. An unusual outbreak occurred in Asian short-clawed otters. Non-specific, neurological, gastrointestinal, respiratory signs and sudden death dominated clinically, mainly in animals over 12 months of age that presented with systemic disease. Pallas’ cats showed encephalitis (inflammation of the brain) and lymphoid tissue was frequently involved in RTLs. Lesions were primarily found in heart, liver, lungs, brain, spleen and lymph nodes. Cases occurred year-round, with species-specific peaks and increases between August and November. This study demonstrates that toxoplasmosis is a significant cause of disease in zoo animals. Knowledge of species susceptibility will aid the treatment of affected animals by zoo veterinarians. Due to the lifecycle of the parasite, feral cat/rodent control is crucial to reduce infection pressure and prevent disease.

**Abstract:**

*Toxoplasma gondii* is an extremely successful zoonotic protozoan parasite that has been demonstrated in a wide range of endo- and poikilothermic species. Although infection is widespread amongst domestic animals, overt disease other than abortion in small ruminants is sporadic. This survey evaluates toxoplasmosis in zoo animals based on a systematic review of pathology archive material (*n* = 33,506 submissions) over a 16-year study period. A total of 126 submissions, deriving from 32 zoos, two educational facilities and two private owners, were included in the study, based on gross lesions, cytological, histological and immunohistological diagnosis of toxoplasmosis. Clinical history, signalment, annual distribution and post-mortem findings were evaluated. A total of 31 species (mammalian 97%/avian 3%) were represented in the study material. Ring-tailed lemurs, slender tailed meerkats, Pallas’ cats, and squirrel monkeys were most affected. An unusual outbreak occurred in Asian small-clawed otters, in which toxoplasmosis has not been reported to date. Clinically, animals over 12 months of age presented with non-specific symptoms (anorexia, weight loss, lethargy, debilitation), neurological, gastrointestinal or respiratory signs and sudden death. Systemic disease predominated, with a propensity for encephalitis in meerkats and Pallas’ cats and systemic disease involving lymphoid tissues in ring-tailed lemurs. Cases in the UK occurred year-round, with species-specific peaks and increases between August and November. This study reinforces the importance of toxoplasmosis as a significant cause of sporadic and epizootic mortalities in a wide range of zoo animals. Feral cat control is crucial to reduce infection pressure.

## 1. Introduction

*Toxoplasma gondii*, the causative agent of toxoplasmosis, is an obligate intracellular protozoan parasite that infects a wide range of endothermic species, including humans. It is present in most regions of the world; environments with a hot, humid climate and lower altitude are favourable for oocyst survival [1]. Members of the family Felidae are the only known definitive hosts for *T. gondii*. Sexual replication occurs in the feline intestine, with shedding of oocysts and sporulation in the environment. Upon ingestion by an intermediate host, oocysts release sporozoites, which undergo asexual replication in intestines and associated lymph nodes, leading to the production of tachyzoites with subsequent migration via the bloodstream to a range of host tissues. Following division cycles, tissue cysts containing bradyzoites form. Cats shed oocysts following uptake of any of the three infectious stages (tachyzoites, bradyzoites, sporozoites). Ingestion by the intermediate host is facilitated by contaminated food or water, uncooked meat, or congenital infection via the transplacental spread of tachyzoites. Intermediate hosts are dead-end hosts, unable to shed oocysts [2]. A range of mammals and birds can serve as intermediate hosts, and this extremely successful parasite has also been demonstrated in aquatic and terrestrial poikilotherms [3,4]. Clinical cases of toxoplasmosis are sporadic in domestic species, except for congenital infections in small livestock ruminants, resulting in abortion and neonatal mortality, which is also the most significant form of the disease in humans. Amongst non-domestic animals, clinical toxoplasmosis has been reported in a wide range of species. In zoos, the occurrence of feral cats and shedding of *T. gondii* oocysts in wild felid faeces can lead to infections in a range of species, amongst which New World primates, prosimians, Australian marsupials and Pallas’ cats (*Otocolobus manul*) have been identified as highly susceptible. Old World monkeys, rats, cattle, and horses seem highly resistant to infection [1,5,6].

This study provides a comprehensive large-scale retrospective review of toxoplasmosis in zoo animals, based on 16 years of pathology archive material from the International Zoo Veterinary Group (IZVG). IZVG is a large, freelance zoo veterinary practice catering for the specialised veterinary needs of zoological collections, mainly in the UK and Europe, and includes a dedicated pathology division. The aim of this study was to identify key susceptible species within the submission material, derived primarily from European zoos, compare the findings to the literature, evaluate clinical symptoms and history, identify key target organs and cause of death across species, assess seasonality, and highlight outbreaks and unusual presentations.

## 2. Materials and Methods

A retrospective database review of submissions received between March 2003 and May 2019 (*n* = 33,506) was undertaken. Cases were included based on necropsy, cytology, histology, and (where applicable) retrospective immunohistochemistry (IHC) findings. Diagnoses were made by board-certified or equivalent veterinary pathologists specializing in zoo and wildlife species (D.D. and M.F.S.), based on cytological and/or histological evidence of *Toxoplasma* organisms in combination with typical pathological lesions (species dependent). Gross post-mortem examination and histology processing followed standard protocols. Impression smear cytology was routinely stained with Leishman’s stain, histology slides with haematoxylin and eosin. Case material for confirmatory IHC was available for 108/126 cases. Immunohistochemistry was performed following the Standard Operating Procedures of the Veterinary Pathology Departments of the Universities of Liverpool (UoL), UK and Munich (LMU), Germany, respectively. Antibodies utilised were rabbit anti-human *Toxoplasma gondii* polyclonal antibody, MyBioSource, MBS373041 (UoL) and VMRD *Toxoplasma gondii* polyclonal antiserum, caprine origin, 210-70-TOXO (LMU). Information, including method of diagnosis, collection of origin, species, signalment, clinical history, gross and histological lesions, affected organs, and concurrent pathological findings, was extracted and analysed using pivot tables (Excel). For the study, age categories were determined as unknown (no age provided), days to <2 months, ≥2−12 months, and >12 months. A Pearson’s chi-squared test was undertaken (Microsoft Excel for Microsoft 365 MSO 32-bit, Microsoft Corporation Redmond, Washington, DC, USA) to determine statistical significances for the sex distribution in the most frequently affected species (ring-tailed lemurs, slender tailed meerkats, Pallas’ cats and squirrel monkeys), excluding cases of unknown sex. To statistically analyse trends over time, Mann–Kendall tests were performed (Real Statistics Rel 7.10, Microsoft Excel). The significance of *Toxoplasma*-related lesions (cause of death or secondary finding) was interpreted based on clinical information, tissue distribution, severity, and absence/presence of concurrent pathology. *Toxoplasma* prevalence in the study population was calculated for each species as the ratio of number of toxoplasmosis cases and total number of submitted cases per species during the study period.

## 3. Results

### 3.1. Case Material

A total of 126/33506 (0.38%) submissions were included in the study; cases occurred between January 2004 and April 2019. A total of 18/126 cases were submitted for complete post-mortem examination. Diagnosis was achieved based on the following combinations: gross post-mortem findings (GPM), cytology and immunohistology (IHC) (3/18), GPM, cytology, histology and IHC (1/18), and GPM, histology and IHC (13/18). For a total of 108 cases, multiple tissues fixed in formalin were received, and diagnosis was achieved by histology in combination with IHC (91/108), clinical *Toxoplasma* titre and histology (7/108) or histology in conjunction with species and clinical history (11/108) (see Appendix A). Submissions were derived from 32 zoos or wildlife parks (*n* = 114), two educational facilities (*n* = 2) and two private owners (*n* = 2). In eight cases, the collection of origin was unknown. Case material included 122 mammal (97%) and 4 bird (3%) cases, from eight orders incorporating 31 species. Mammals belonged to the orders Primates (*n* = 58), Carnivora (*n* = 53), Rodentia (*n* = 5), Diprotodontia (*n* = 4), Artiodactyla (*n* = 1), and Dasyuromorphia (*n* = 1). Avian species were exclusively in the orders Coliiformes (*n* = 3) and Musophagiformes (*n* = 1). On a species level, ring-tailed lemurs (*Lemur catta*) (*n* = 31), slender-tailed meerkats (*Suricata suricatta*) (*n* = 30), Pallas’ cats (*Otocolobus manul*) (*n* = 13), and common squirrel monkeys (*Saimiri sciureus*) (*n* = 13) were overrepresented, with respective prevalences of 28.4% (31/109), 18.3% (30/164), 68.4% (13/19), and 10.6% (13/123) within the species populations. Enhanced details are provided throughout the manuscript for these highly susceptible species. Small outbreaks or clustered individual cases occurred in Asian small-clawed otters (*Aonyx cinereus*) (*n* = 5), Patagonian maras (*Dolichotis patagonum*) (*n* = 5), speckled mousebirds (*Colius striatus*) (*n* = 3), emperor tamarins (*Saguinus imperator*) (*n* = 2), and pied tamarins (*Saguinus bicolor*) (*n* = 2). Single cases were identified in an alpaca (*Vicugna pacos),* black-and-white ruffed lemur (*Varecia variegata* *variegata*), black-capped squirrel monkey (*Saimiri boliviensis*), black-footed cat (*Felis nigripes*), cheetah (*Acinonyx jubatus*), common marmoset (*Callithrix jacchus*), crowned sifaka (*Propithecus coronatus*), Eurasian badger (*Meles meles meles*), Hartlaub’s turaco (*Tauraco hartlaubi*), kowari (*Dasyuroides byrnei*), potoroo (*Potorous sp*.), pygmy slow loris (*Nycticebus pygmaeus*), red-backed bearded saki (*Chiropotes chiropotes*), red-handed tamarin (*Saguinus midas*), red-necked wallaby (*Macropus rufogriseus*), red panda (*Ailurus fulgens*), snow leopard (*Panthera uncia*), tarsier (*Tarsius sp*.), titi monkey (*Callicebus cupreus*), western grey kangaroo (*Macropus fuliginosus*), woolly monkey (*Lagothrix lagotricha*), and yellow-footed rock-wallaby (*Petrogale xanthopus*).

### 3.2. Age Distribution

**Figure 1:**
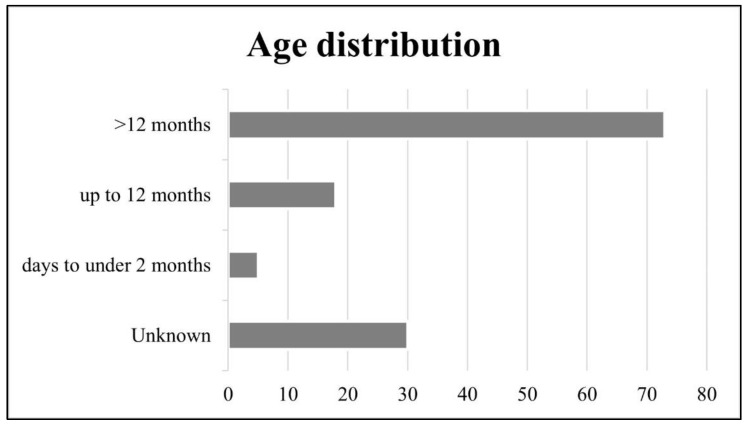
Overview of affected ages. Infection is most common in animals over 12 months of age.

Affected animals ranged in age from neonate to adult. An overview of age distribution is provided in Figure 1. Toxoplasmosis was most common in animals over 12 months of age (73/126). Disease in animals ranging from days to <2 months and ≥2−12 months was seen in slender tailed meerkats (*n* = 2; *n* = 8, respectively), Pallas’ cats (*n* = 2; *n* = 3, respectively), Asian small-clawed otters (≥2−12 months *n* = 4), squirrel monkeys (≥2−12 months *n* = 2) and a neonate pygmy slow loris (*n* = 1).

### 3.3. Sex Distribution

The overall study population comprised 71 males and 42 females; the sex was unknown in 13 cases. Sex distribution was comparatively equal in squirrel monkeys (6/13 males, 5/13 females, 2 unknown). More females were seen amongst Pallas’ cat cases (9/13, 69%) and more males amongst slender-tailed meerkat (18/30, 60%) and ring-tailed lemurs (21/31, 68%) submissions. However, the only significant sex difference in toxoplasmosis cases was recorded for ring-tailed lemurs, in which females were more likely to be diagnosed (21 females/31 total submissions, 68%; *p* < 0.05, Pearson’s chi-squared test).

### 3.4. Clinical Presentation

**Figure 2:**
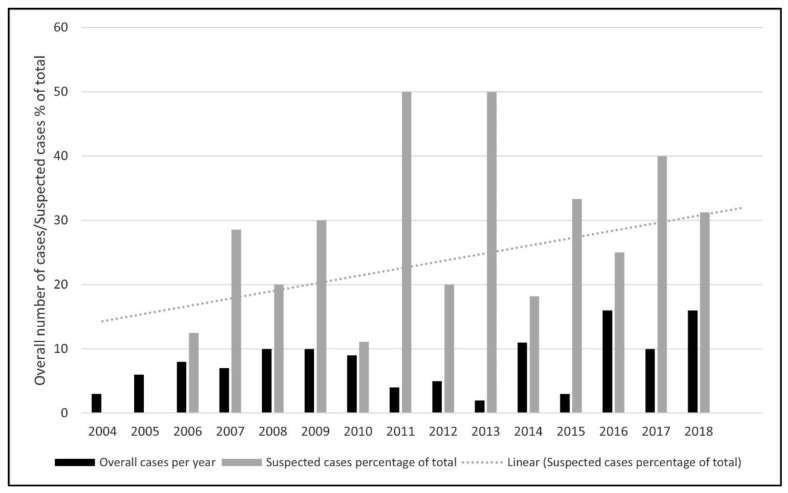
Pale grey dotted line indicates a statistically significant rising annual trend in the per-centage of total cases submitted with a clinical suspicion of toxoplasmosis (*p* < 0.05, Mann–Kendall test, Real Statistics Rel 7.10, Microsoft Excel). There is no rising trend in the overall number of confirmed toxoplasmosis cases over the same period (*p* > 0.05, Mann–Kendall test).

Clinical presentation based on information provided by the submitting veterinarian or institution is provided in Table 1. Neurological presentation dominated in meerkat (13/30) and Pallas’ cat (8/13) populations. Ring-tailed lemurs presented mostly with non-specific (16/31) or gastrointestinal (11/31) signs, and squirrel monkeys with a history of sudden death (6/13) in the absence of clinical symptoms. Based on available submission information, toxoplasmosis was not suspected as the cause of death in 73% of all submitted cases. In 23% of submissions, *Toxoplasma* was queried by the submitting collection or veterinarian or listed as the suspected cause of death. In 4% of cases, insufficient history was available. These data, analysed over time, suggested an increase in suspected case numbers towards the end of the study period (2016–2018) (Figure 2). For further confirmation of a trend over time, a Mann–Kendall test was performed (Real Statistics Rel 7.10, Microsoft Excel), utilizing total number of cases versus the percentage of cases with a clinical suspicion of toxoplasmosis based on the submission information. This revealed an increasing trend over time in the percentage of cases submitted with a clinical suspicion of toxoplasmosis (*p* < 0.05, Mann–Kendall test).

### 3.5. Cause of Death

Based on a pathological examination evaluating organ distribution and associated tissue changes, lesion severity, and the absence/presence of concurrent disease processes, toxoplasmosis was considered the cause of death in 92% of animals included in the study (116/126). Further detail at species level is included below and in Table 2.

### 3.6. Post-Mortem Findings

**Figure 3:**
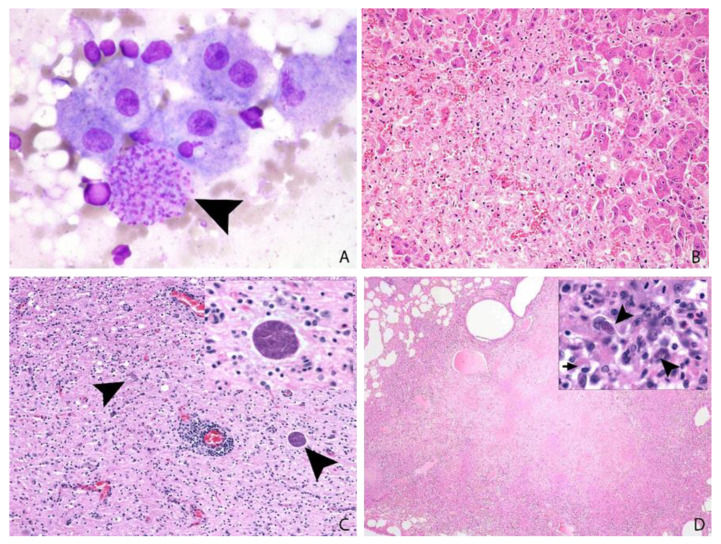
(**A**,**B**): Slender-tailed meerkat, 3 years, female, liver. A: Amongst hepatocytes, histiocytes and a background of erythrocytes there is a large cluster of intracytoplasmic *Toxoplasma* tachyzoites (arrow head); impression smear cytology, Leishman’s stain, ×100. (**B**): Large areas of coagulative necrosis efface hepatic parenchyma. Organisms are difficult to identify: H&E, ×20. (**C**): Pallas’ cat, 9 years, female, brain: Moderate to marked mixed mononuclear encephalitis with perivascular cuffing and intralesional protozoa. H&E, ×10, inset ×60. (**D**): Eurasian badger, adult, female, lung: Severe coalescent necrotising pneumonia. Intralesional intracellular tachyzoites (arrow heads) are present, and the cytoplasm of macrophages contain intralesional bacteria (arrow) (identified as mycobacteria on special stains). H&E, ×2, inset ×100.

**Figure 4:**
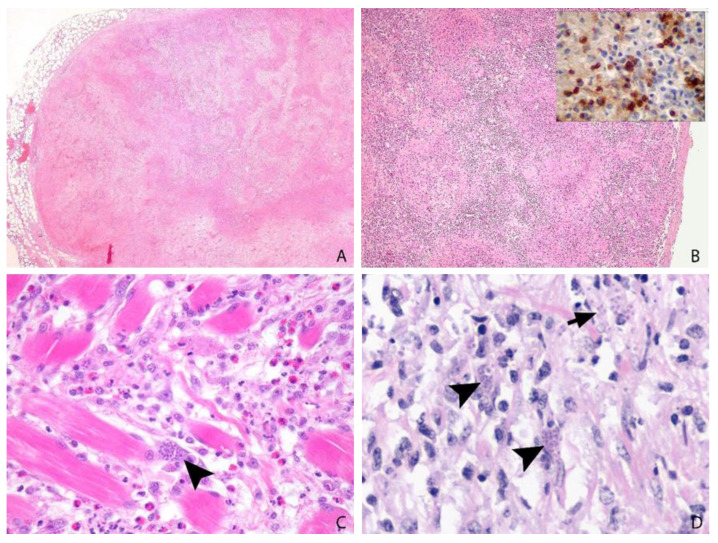
(**A**): Ring-tailed lemur, 2 years, male, mesenteric lymph node. Severe confluent necrotising lymphadenitis. H&E, ×2. (**B**): Speckled mousebird, >7 years, male, spleen. Multifocal to coalescing necrotising splenitis with myriads of intralesional *T. gondii* protozoa, confirmed by immunohistochemistry (inset). H&E, ×2 and IHC, ×60. (**C**): Snow leopard, 18 years, female, tongue. Glossal myofibres are separated by histiocytic and lesser neutrophilic infiltrates, amongst which protozoal tachyzoites (arrow head) are identified. (**D**): Alpaca, 14 years, male, abdominal mass. Numerous clusters of intracellular (arrow heads) and extracellular (arrow) tachyzoites are present amongst sheets of mononuclear inflammation, embedded on fibrovascular stroma. H&E, ×60.

In the 18 cases submitted for complete gross post-mortem examination (ring-tailed lemur *n* = 10, common squirrel monkey *n* = 4, slender tailed meerkat *n* = 3 and crowned sifaka *n* = 1), gross findings comprised hepatitis and lymphadenitis (12/18), pneumonia (9/18), splenomegaly (8/18), icterus (4/18), myocarditis, thoracic effusions, enteritis (2/18), and pancreatitis (1/18). Two animals showed no significant gross lesions. The cytological or histological identification of characteristic 2–6 µm, round- to crescent-shaped, basophilic protozoal tachyzoites and/or approximately 20 µm × 15 µm tissue cysts characterised by a thin wall encircling numerous 1–2 µm, elongate bradyzoites in association with necrosis and variable inflammation in one or more organs is highly suggestive of toxoplasmosis (Figure 3 and Figure 4) [7]. When sparse, intralesional organisms can be difficult to demonstrate and IHC is often helpful in suspect cases (Figure 4B). *Neospora caninum*, which can be differentiated from *Toxoplasma* by IHC or electron microscopy, is the most important differential diagnosis.

### 3.7. Tissue Distribution

**Figure 5:**
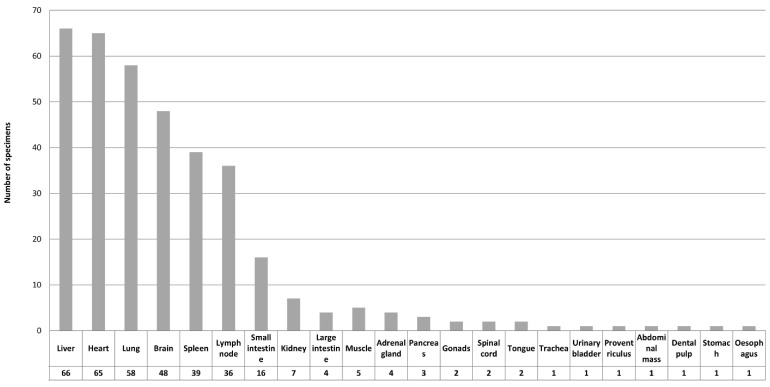
*Toxoplasma*-related lesions were evident in a wide range of tissues, presented in descending order.

**Figure 6:**
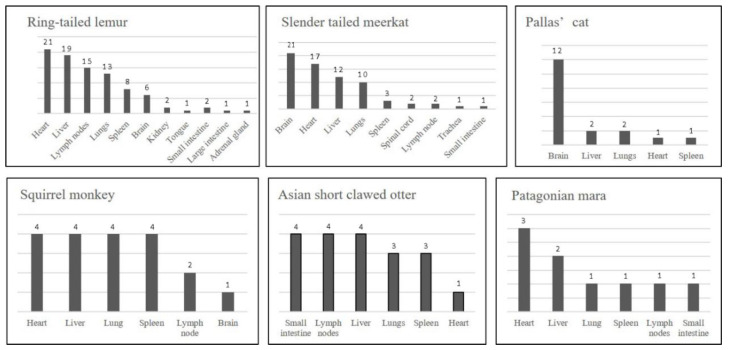
Tissue distribution of *Toxoplasma* lesions in ring tailed lemurs (*n* = 31), slender tailed meerkats (*n* = 30), Pallas’ cats (*n* = 13), squirrel monkeys (*n* = 13), Asian small-clawed otters (*n* = 5) and Patagonian maras (*n* = 5). The Y axis values demonstrate the frequency of *Toxoplasma*-induced lesions.

In the study material, *Toxoplasma* lesions were identified in a wide range of tissues, an overview of which is provided in Figure 5. Liver, heart, lung, brain, lymphoid tissues, and small intestine were identified as the most significant target organs. Species-related differences are exemplified in Figure 6.

Ring-tailed lemurs presented with systemic toxoplasmosis. *Toxoplasma* lesions were identified in heart (*n* = 21), liver (*n* = 19), mesenteric lymph nodes (*n* = 15), lung (*n* = 13), spleen (*n* = 8), brain (*n* = 6), kidney (*n* = 2), small intestine (*n* = 2), large intestine (*n* = 1), adrenal gland (*n* = 1), and tongue (*n* = 1). Toxoplasmosis was considered the cause of death in 31/31 animals. Consistent with the clinical presentation, lesions were most commonly detected in the brain (*n* = 21), but also in the heart (*n* = 17), liver (*n* = 12), lung (*n* = 10), spleen (*n* = 3), lymph node (*n* = 2), spinal cord (*n* = 2), small intestine (*n* = 1), and trachea (*n* = 1) of slender tailed meerkats. Toxoplasmosis was the cause of death in 29/30 cases. In Pallas’ cats, the brain was most affected (*n* = 12); other affected organs included the lung (*n* = 2), liver (*n* = 2), heart (*n* = 1), and spleen (*n* = 1). In common squirrel monkeys, lesions were found in the heart (*n* = 4), lung (*n* = 4), liver (*n* = 4), spleen (*n* = 4), lymph node (*n* = 2), and brain (*n* = 1). Toxoplasmosis was considered the cause of death in 13/13 Pallas’ cats and 13/13 squirrel monkeys. A single outbreak of toxoplasmosis was recorded in Asian small-clawed otters in November 2018, affecting only juveniles (*n* = 5) from one collection. *T. gondii* infection was not suspected clinically in animals presenting with sudden death, gastrointestinal signs, and body cavity effusions. Pathology revealed lesions in small intestine (*n* = 4), liver (*n* = 4), lymph node (*n* = 4), lung (*n* = 3), spleen (*n* = 3), and heart (*n* = 1). All five animals were considered to have died due to toxoplasmosis. Affected Patagonian maras showed lesions in the heart (*n* = 3), lung (*n* = 3), liver (*n* = 2), spleen (*n* = 1), lymph node (*n* = 1), and small intestine (*n* = 1). Toxoplasmosis was considered the cause of death in 5/5 animals. Further details for remaining species (*n* < 4) are summarized in Table 2.

### 3.8. Annual Distribution

**Figure 7:**
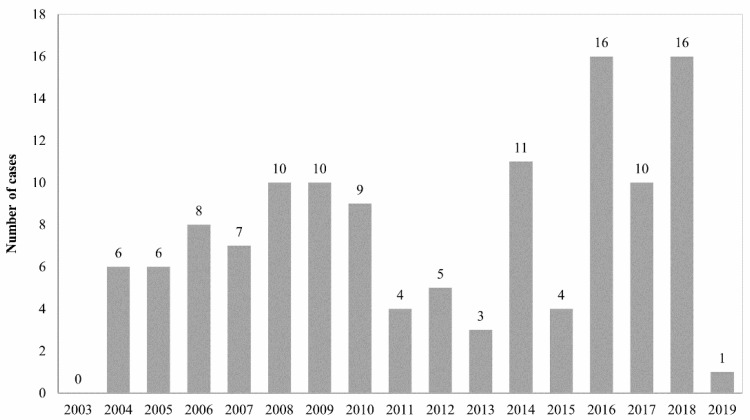
Annual distribution of case numbers between March 2003 and May 2019.

**Figure 8:**
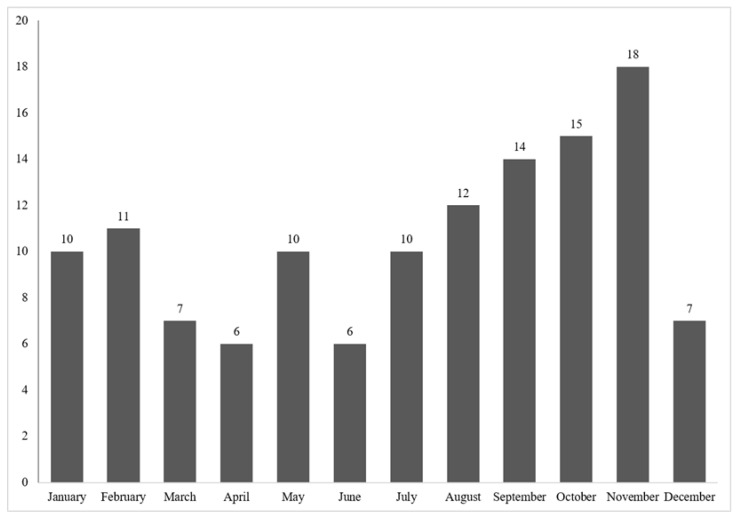
Monthly distribution of case numbers over the study period.

**Figure 9:**
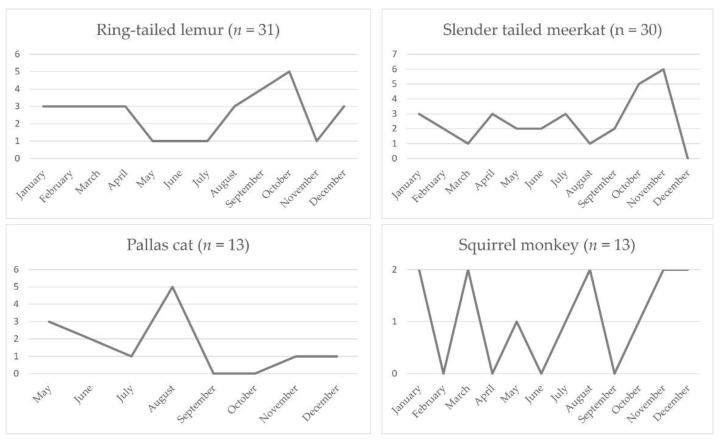
Monthly distribution of case numbers in ring-tailed lemurs, slender-tailed meerkats, Pallas’ cats, and squirrel monkeys. Pallas’ cat cases were not recorded between January and April.

The annual and monthly case distribution during the study period is outlined in Figure 7 and Figure 8. Case numbers ranged from 0 cases in 2003 to 16 cases in 2016 and 2018, respectively. Across all species, cases occurred year-round, while most cases were submitted between August and November (Figure 8). The monthly distribution varied between species (Figure 9). Ring-tailed lemur cases spiked in September and October, slender-tailed meerkat cases in October and November. Most Pallas’ cat cases occurred in August, whilst 0–2 squirrel monkey cases were submitted from January through to December.

## 4. Discussion

*Toxoplasma gondii* is one of the most polyxenous parasites that is known and distributed worldwide. Though toxoplasmosis in zoo animals has been recognised as a significant cause of sporadic and epizootic mortalities and is implicated as an important threat to some endangered species, large-scale pathology studies have not been undertaken to date [4,6,8,9,10,11,12,13,14,15,16,17]. The current study reviews findings in 126 *Toxoplasma* cases across 31 mammalian and avian species over a 16-year study period.

Species that are overrepresented in the study material include ring-tailed lemurs, slender-tailed meerkats, Pallas’ cats, and common squirrel monkeys. A high susceptibility for toxoplasmosis has previously been reported in New World primates, prosimians, meerkats and Pallas’ cats [8,13,14,15,18,19,20]. It is likely that this is due to evolutionary novel exposures, as these species have largely evolved separately from *T. gondii*. This is supported by serological studies in wild Pallas’ cats, Tasmanian wallabies and ring-tailed lemurs, highlighting a very low seroprevalence in native habitats [21,22,23]. Whilst Australian marsupials, particularly macropods, wombats and bandicoot, as well as marine mammals, are also considered highly susceptible species, very few cases were recognised in marsupials in our submission material, and no cases were identified in marine mammals. Over the study period, >200 Australian marsupial submissions (all macropods) and >450 marine mammal submissions were received; however, toxoplasmosis was only recognised in a red-necked wallaby, western grey kangaroo, potoroo and yellow-footed rock wallaby (*n* = 4). Reports of toxoplasmosis in European captive macropod populations are also rare, in contrast to studies in Australia and findings in zoos in the United States [24,25]. The cause of this discrepancy is unclear. Inadequacies in the submission material are possible. In a subset of submissions, the submitted materials would not have been suitable to confirm or refute possible toxoplasmosis, for example, in cases with a limited tissue selection or single-tissue biopsies. It could also be speculated that differences in the husbandry, climate or density of feral cat populations between European and other collections, resulting in a reduction in infection pressure, may be implicated. In addition, it has been demonstrated that exposure to *T. gondii* is not invariably fatal. Disease development may be influenced by the virulence and strain of *T. gondii* involved, as well as the inoculation dose and immune status of the host [26]. No investigation of toxoplasmal strains was possible as part of this retrospective study.

Co-infection with canine distemper virus has been associated with toxoplasmosis in grey foxes and a captive snow leopard [27,28,29]. It is feasible that immunosuppressive diseases, including a range of viral infections, or neoplasia such as lymphoma, could predispose to systemic toxoplasmosis. In the present study, a comprehensive histological evaluation of over 120 cases did not yield evidence of significant co-infections or concurrent neoplastic disease.

In November 2018, an outbreak of systemic toxoplasmosis occurred on the Isle of Man in a group of Asian small-clawed otters, a species for which no published reports of toxoplasmosis could be identified. Animals presented with sudden death, wobbling, gastrointestinal signs and body cavity effusions. On pathological examination, systemic disease was dominated by enteritis, lymphadenitis, and pneumonia in juvenile animals. Deaths occurred in short succession in a group of 14 juveniles following the construction of an extension pen, catch-up and anaesthesia for pre-transport assessment. Toxoplasmosis had been seen in squirrel monkeys in an adjacent enclosure attributed to feral cats, and a fishing cat was also housed nearby. Wallabies are present in the collection and roam free on the island, which also has a significant small ruminant population. The collection has many waterways and heavy rain had occurred prior to the outbreak. The definitive origin of infection could not be determined, but it is highly likely that the outbreak relates to a high oocyst burden in the environment, maintained by felids and a range of susceptible intermediate hosts species or vector hosts. Recent flushing of the water system, stress due to building work, catch-up and anaesthesia may have been contributory factors.

Three cases of systemic toxoplasmosis were identified in speckled mousebirds and confirmed by immunohistochemistry (Figure 4B). Clinical toxoplasmosis has been reported from a range of avian species including pigeons and canaries (severe clinical disease), Hawaiian crows, bowerbirds, psittacines, galliformes, penguins and a speckled mousebird [30,31], but there is considerable confusion amongst many pathologists regarding the identity of *T. gondii*-like parasites and the diagnosis of toxoplasmosis in avian species. Care must be taken to avoid misdiagnosis of haemoprotozoa, such as systemic isosporosis (“atoxoplasma”) and *Sarcocystis* species, which are common in passerines (both) and psittacines (*Sarcocystis*).

One serological study demonstrated a low prevalence of toxoplasmosis in alpacas, and abortion can occur [32,33]. In this study, one case of toxoplasmosis was seen in an alpaca presenting with an abdominal mass, suspected to be a neoplasm. Sections of the mass only were submitted and revealed granulation tissue with extensive areas of coagulative necrosis and myriads of intralesional protozoal tachyzoites consistent with toxoplasmosis (Figure 4D). Toxoplasmal lymphadenitis or pancreatitis could have been implicated in this case; however, no pre-existing tissue was identified in the submitted sections, and no further clinical information was available.

Serum antibodies to *T. gondii* could be detected in 20% of wild snow leopards in Mongolia, included in a serological survey study, without overt clinical disease [34]. Active toxoplasmosis in a mesenteric lymph node could also be demonstrated in a captive snow leopard with concurrent canine distemper infection [29]. A case of glossal toxoplasmosis in an 18-year-old male snow leopard that presented with a fibrous mass in the tongue base was included in this study. The animal had received low-dose steroids for a skin condition and was euthanased after a period of apathy and drooling associated with the tongue lesion. Histology confirmed neutrophilic and histiocytic glossitis with numerous intralesional tachyzoites (Figure 4C). Focal recrudescence of infection from a tissue cyst in the tongue muscle, possibly related to immunosuppression from the period of steroid treatment, was hypothesized, as such focal lesions are occasionally seen in immune-suppressed older domestic cats [35].

Most animals in our study were over 12 months of age (73/126) without evidence of concomitant disease, in contrast to presentations in domestic species, in which systemic fatal toxoplasmosis occurs most often in young animals, especially immunologically immature neonates, and in immunocompromised hosts. Toxoplasmosis should be considered in juvenile and subadult meerkats, Asian small-clawed otters and squirrel monkeys. It is known to be a possible cause of fulminant systemic disease in Pallas’ cat neonates and was found in a pygmy slow loris neonate in our study.

Proportionately more female Pallas’ cats and more male slender-tailed meerkats and ring-tailed lemurs (in the latter statistically significant) were affected. It could be speculated that the infection pressure may be higher for female than male Pallas’ cats, as they attend to their offspring. Studies in humans identify the male gender as a risk factor for seropositivity to *T. gondii*, which was correlated with eating habits (male Germans eat about twice as much meat and meat products as females) [36]. It is feasible that eating and/or other habits, which are variable between sex, may also play a role in captive meerkat and lemur populations.

A range of clinical presentations was recorded in our study, dominated by non-specific clinical symptoms including anorexia, depression, lethargy, weight loss, collapse, debilitation, dehydration. A neurological presentation in meerkats and Pallas’ cats, including adults, should raise suspicion of toxoplasmosis in these species. Gastrointestinal signs were evident in ring-tailed lemurs and *Toxoplasma* should be included as a differential diagnosis for a gastrointestinal presentation in this and other susceptible species (for example, Asian small-clawed otters). Parasitological examination of faeces cannot detect the organism, as oocysts are not shed by intermediate hosts. Therefore, *Toxoplasma* serology should be included in the clinical work-up of suspected cases. PCR analysis of whole blood could also be explored.

It is recognised that the dissemination of *Toxoplasma* in the host occurs in lymphocytes, macrophages, granulocytes, and freely in plasma. From the intestine the organisms can take two routes: via lymphocytes to regional nodes, into the lymph and bloodstream, or via the portal circulation to the liver and from there to the systemic circulation, from which a wide variety of organs can be reached. The hallmarks of systemic toxoplasmosis are interstitial pneumonia, hepatitis, lymphadenitis, myocarditis, and nonsuppurative meningoencephalitis [35]. Our study identified liver, heart, lung, brain, lymphoid tissues, and small intestine as the most significant target organs in the study population. Species-specific differences could be recorded, influencing the tissue sampling recommendations outlined for key susceptible species in Table 3.

Although cases were observed all year-round, submissions peaked between August and November, and variations were evident between species. Climatic factors such as increased temperature and humidity create favorable environments for oocyst survival, resulting in an increase in infection pressure, which may play a role. Seasonal reproductive patterns and associated stressors may also correlate. Ring-tailed lemurs, for example, mate around April; all breeding females in a group mate within a few weeks, resulting in births between August and September. This pattern appears to be closely followed by the observed seasonal distribution of *Toxoplasma*. Pallas’ cats typically breed between December and early March, with a gestation period lasting for an average of 75 days, also coinciding with the observed case distribution. Other husbandry factors and social dynamics, as well as the breeding and social activities of feral cats, should also be considered as possible contributing factors.

In our study, toxoplasmosis was queried or suspected in 23% of cases, but considered the cause of death in 92% of animals included in the study. The timeline review of cases, and a statistically significant rising annual trend in the percentage of total cases submit-ted with a clinical suspicion of toxoplasmosis (Figure 2), suggests an increasing level of veterinary/curatorial suspicion for toxoplasmosis in cases submitted towards the end of the study period. Although there could be multiple contributory factors, this might demonstrate the educational value of regular pathology surveillance as part of staff training.

## 5. Conclusions

In summary, to the authors’ knowledge, this study provides the largest scale pathology review of toxoplasmosis in zoo species to date, based on its wide geographical and species coverage and case selection from more than 30 captive management facilities. This study confirms that exposure to toxoplasmosis is a significant cause of sporadic and epizootic mortalities and threatens a range of endangered species. It highlights outbreaks and individual cases in species not previously recorded in the veterinary literature. Histological diagnosis is reliant on the submission of relevant tissue sets and care should be taken to avoid misdiagnosis of *Toxoplasma* versus haemoparasites in birds. Feral cat control is crucial to reduce infection pressure, but further clinical studies to evaluate additional protective measures for highly susceptible species are also indicated, taking predisposing factors into account.

## Figures and Tables

**Table 1:** Clinical presentation. Non-specific and neurological symptoms and a history of sudden death dominate the clinical picture.

Clinical Presentation/Organ System	Number of Animals
Non-specific (anorexia, depression, lethargy, weight loss, collapse, debilitation, dehydration)	37
Neurological	30
Sudden death/found dead	28
Gastrointestinal/hepatic (incl. jaundice)	24
Respiratory	17
Body cavity effusions	11
Urinary	4
Cardiac	4
Abdominal mass	2
Abortion	1
Special senses	1
Insufficient history	12

**Table 2:** Overview of signalment, clinical presentation, affected tissues and *Toxoplasma*-related death in species with *n* < 4.

Common Name	Age	Sex	Clinical Signs	Affected Tissues	Cause of Death
Alpaca	14 years	Male	Abdominal mass	Abdominal mass	No
Black-and-white ruffed lemur	20 years	Female	Gastrointestinal/hepatic, respiratory	Heart	No
Black-capped squirrel monkey	4 years	Female	Sudden death/found dead, body cavity effusions	Liver, spleen, lymph node, small intestine	No
Black-footed cat	Adult	Male	Non-specific, gastrointestinal/hepatic, urinary	Heart	No
Cheetah	6 months	Male	Non-specific, gastrointestinal/hepatic, respiratory	Heart, lung, spleen, small intestine, large intestine, pancreas, urinary bladder	No
Common marmoset	2 years	Male	Neurological, non-specific	Lung, liver	Yes
Crowned sifaka	9 years 6 months	Female	Non-specific, gastrointestinal/hepatic	Heart, lung, liver, spleen, lymph node	Yes
Emperor tamarin (*n* = 2)	3 months, adult	Male	Insufficient	Heart, lung, liver	Yes
Eurasian badger	Adult	Female	Neurological, non-specific, abdominal mass	Lung	Yes
Hartlaub’s turaco	14 years	Male	Sudden death/found dead	Muscle	No
Kowari	Adult	Female	Neurological	Brain	Yes
Pied tamarin (*n* = 2)	9 years, 4 years 6 months	Male/Female	Respiratory	Lung, liver, spleen, lymph node, adrenal gland, small intestine, large intestine, heart	Yes
Potoroo	6 years	Male	Insufficient	Brain	Yes
Pygmy slow loris	Neonate	Unknown	Abortion	Heart, kidney, brain, muscle, dental pulp	Yes
Red panda	Adult	Male	Non-specific	Liver, spleen, lymph node	Yes
Red-backed bearded saki	2 months	Female	No history provided	Heart, lung, liver, spleen	Yes
Red-handed tamarin	Adult	Male	Gastrointestinal/hepatic	Lung, liver, lymph node	Yes
Red-necked wallaby	Juvenile	Female	No history provided	Lung, liver, lymph node, small intestine	Yes
Snow leopard	18 years	Male	Gastrointestinal/hepatic, urinary	Tongue	No
Speckled mousebird (*n* = 3)	Juvenile, Adult (*n* = 2)	Male (*n* = 2), unknown	Sudden death	Heart, liver, lung, spleen, brain, kidney, gonad, pancreas, adrenal gland, proventriculus, small intestine, muscle	Yes
Tarsier	Adult	Male	Neurological	Muscle	No
Titi monkey	5 years 9 months	Female	Non-specific, sudden death/found dead	Heart, lung, liver, lymph node	Yes
Western grey kangaroo	3 years	Male	Sudden death/found dead	Lymph node	Yes
Woolly monkey	14 years	Male	Gastrointestinal/hepatic, respiratory	Lung, spleen	Yes
Yellow-footed rock-wallaby	Adult	Unknown	Insufficient	Heart, lymph node, small intestine, large intestine, kidney, muscle, stomach, oesophagus	Yes

**Table 3:** Species-specific recommendations for minimum sampling requirements in suspect toxoplasmosis cases.

Common Name	Recommended Minimum Tissue Selection for Histology
Ring-tailed lemur	heart, liver, mesenteric lymph nodes, lung, spleen, brain
Slender tailed meerkat	brain, heart, liver, lung
Pallas’ cat	brain, liver
Common squirrel monkey	heart, lung, liver, spleen

## Data Availability

Datasets used are derived from the database of the International Zoo Veterinary Group. As client, clinical and pathology data are confidential, detailed datasets are not publicly provided.

## Appendix A

For 11 animals, material for retrospective immunohistology was not available at the time of the study. The authors decided to include these animals based on the following reasoning: whilst the authors appreciate that *Neospora* species have structural similarities that hinder definitive differentiation of these two parasites in histology, *Neospora* infection is highly unlikely in zoo species, contrary to *Toxoplasma* infection, which is highly likely. This rational is further supported by the different life cycle of *Toxoplasma* versus *Neospora* parasites, with zoo species being regularly exposed to feral cat faeces/carrier rodents etc, another well-established problem in a zoo setting that is not applicable to *Neospora* transmission. In addition, the 11 selected cases were all derived from species particularly susceptible to disease, such as Pallas’ cats, slender-tailed meerkats and ring-tailed lemurs. Several of the cases additionally presented with affected conspecifics, for which confirmatory immunohistochemistry was available. In other cases, *Toxoplasma* involvement was further backed up by the subsequent successful treatment of remaining animals in the group.
